# Increased Levels of Leukocyte-Derived MMP-9 in Patients with Stable Angina Pectoris

**DOI:** 10.1371/journal.pone.0019340

**Published:** 2011-04-29

**Authors:** Simon Jönsson, Anna Lundberg, Hanna Kälvegren, Ida Bergström, Aleksander Szymanowski, Lena Jonasson

**Affiliations:** Division of Cardiovascular Medicine, Department of Medical and Health Sciences, Faculty of Health Sciences, Linköping University, Linköping, Sweden; University of Modena and Reggio Emilia, Italy

## Abstract

**Objective:**

There is a growing interest for matrix metalloproteinases (MMPs) and their tissue inhibitors (TIMPs) in plasma as novel biomarkers in coronary artery disease (CAD). We aimed to identify the sources of MMP-8, MMP-9, TIMP-1 and TIMP-2 among peripheral blood cells and further explore whether gene expression or protein release was altered in patients with stable angina pectoris (SA).

**Methods:**

In total, plasma MMP-9 was measured in 44 SA patients and 47 healthy controls. From 10 patients and 10 controls, peripheral blood mononuclear cells (PBMC) and neutrophils were isolated and stimulated *ex vivo*. MMPs, TIMPs and myeloperoxidase were measured in plasma and supernatants by ELISA. The corresponding gene expression was measured by real-time PCR.

**Results:**

Neutrophils were the dominant source of MMP-8 and MMP-9. Upon moderate stimulation with IL-8, the neutrophil release of MMP-9 was higher in the SA patients compared with controls (p<0.05). In PBMC, the TIMP-1 and MMP-9 mRNA expression was higher in SA patients compared with controls, p<0.01 and 0.05, respectively. There were no differences in plasma levels between patients and controls except for TIMP-2, which was lower in patients, p<0.01.

**Conclusion:**

Measurements of MMPs and TIMPs in plasma may be of limited use. Despite similar plasma levels in SA patients and controls, the leukocyte-derived MMP-9 and TIMP-1 are significantly altered in patients. The findings indicate that the leukocytes are more prone to release and produce MMP-9 in symptomatic and angiographically verified CAD—a phenomenon that may have clinical implications in the course of disease.

## Introduction

Matrix metalloproteinases (MMPs) are a family of extracellular matrix degrading enzymes in which several members, like MMP-8 and MMP-9, are implicated in atherosclerotic plaque development and atherothrombosis [1]–[3]. In patients with coronary artery disease (CAD), MMP-9 in plasma is a predictor of rapid plaque progression [4] and in-stent restenosis [5]. Serum or plasma levels of MMP-8 and MMP-9 are also associated with cardiovascular outcome in patients with an established diagnosis of CAD [6]–[10]. In cross-sectional studies, transient elevations of MMP-8 or MMP-9 have been associated mainly with acute coronary syndrome [11]–[14]. Studies of patients with clinically stable CAD have, on the other hand, shown inconsistent results, in particular for MMP-9. Some have shown increased levels of MMP-8 and MMP-9 in patients with stable angina (SA) when compared with healthy individuals [11]–[13], [15]–[19] while others have not shown any differences [12]–[14], [20].

There may be several cellular sources of MMP-8 and MMP-9 in plasma. Circulating levels are believed to reflect, at least in part, the release of MMPs from cells in the arterial wall, such as smooth muscle cells and macrophages. Peripheral blood cells are another potential source of MMPs in plasma. The higher levels of MMP-8 and MMP-9 in serum compared to plasma are assumed to result from the release by neutrophils during the coagulation process in the serum tube [21], [22]. In neutrophils, MMP-8 and MMP-9 are synthesized during the late stage maturation process in the bone marrow and thereafter stored in specific and gelatinase granules until needed. In particular, MMP-8 is known as the “neutrophil collagenase” [23]. It has also been shown that several MMPs including MMP-8 and MMP-9 are constitutively expressed by the mononuclear cells in peripheral blood [24], [25]. An increased expression and/or release of MMPs have been associated with a primed or activated state of leukocytes and may play a key role for the adhesion and transmigration into the arterial wall.

Tissue inhibitors of metalloproteinase (TIMP)-1 and TIMP-2 are the main endogenous regulators of MMP-8 and MMP-9 activity. An imbalance between TIMPs and MMPs is believed to be crucial for the maintenance of plaque stability and interestingly, reduced amounts of TIMP-1 and TIMP-2 have been reported in human unstable plaques compared to stable plaques [26], [27]. A few clinical studies have shown increased plasma levels of TIMP-1 in SA patients [13], [15] while others have reported levels similar to healthy controls [14], [16], [17]. Similarly, studies regarding the clinical association of circulating TIMP-2 levels have shown contradictory results [13], [15], [16]. According to the literature, cellular sources of TIMP-1 and TIMP-2 may include several cell types, such as smooth muscle cells, macrophages and circulating mononuclear cells [24], [28].

There is a growing interest for MMPs and TIMPs as both diagnostic markers and therapeutic targets in CAD. However, the measurements of plasma levels may be insufficient to detect differences of potential clinical relevance. This has been illustrated in previous studies of CAD patients showing an increased expression of MMP-9 in neutrophils [20] and monocytes [14] without any concomitant changes in plasma levels. The aim of the present study was to identify the sources of MMP-8, MMP-9, TIMP-1 and TIMP-2 among peripheral blood cells. We further hypothesised that the gene expression or protein release was altered in patients with symptomatic and angiographically verified CAD compared with healthy individuals.

## Methods

### Ethics Statement

Written informed consent was obtained from study participants and the research protocol was approved by the Ethical Review Board of Linköping University. The study was conducted in accordance with the ethical guidelines of Declaration of Helsinki (Linköping University M242-08)..

### Subjects

Forty-four SA patients referred for elective coronary angiography at the Department of Cardiology, Linköping University Hospital, Sweden were included in the study. They had clinical evidence of Canadian Cardiovascular Society class II and III [29]and positive exercise tests or myocardial scintigrams. The diagnosis of CAD was angiographically verified in all patients. Exclusion criteria were severe heart failure, immunologic disorders, neoplastic disease, evidence of acute or recent (<2 months) infection, recent major trauma, surgery or revascularization procedure, or treatment with immunosuppressive or anti-inflammatory agents (except low-dose aspirin). Forty-seven clinically healthy age and sex matched controls were randomly selected from a population register representing the hospital recruitment area. None of them were taking any medication. Heparinized venous peripheral blood and serum with activation clot was collected in the morning after a fasting for 12 h and in patients, always prior to coronary angiography.

### Preparation and stimulation of peripheral blood mononuclear cells and neutrophils

For the ex vivo assays, a subgroup of 10 patients was randomly selected from the large group and matched with 10 controls. Peripheral blood mononuclear cells (PBMCs) and granulocytes, predominantly constituted by neutrophils, were separated from heparinised whole blood by density centrifugation as previously described [30]. In short, freshly drawn heparinized blood was layered on top of Lymphoprep™ and Polymorphprep™ (Axis-Shield PoC AS, Oslo, Norway), and centrifuged in a swing-out centrifuge (420×g, 40 min, room temperature (RT)), thus producing one band of PBMCs and one of neutrophils.

The PBMCs were collected and washed in phosphate buffered saline (PBS) with 0.1% fetal bovine serum (FBS) (PAA Laboratories GmbH, Pasching, Austria) by centrifugation (400×g, 10 min, 4°C) twice. The cells were resuspended in RPMI-1640 media supplemented with L-glutamine (Gibco by Invitrogen, Carlsbad, CA, USA), 10% FBS, 100 U/ml penicillin and 100 µg/ml streptomycin (Gibco by Invitrogen, Carlsbad, CA, USA) to a concentration of 5×10^6^ cells/ml. PBMCs were stimulated with phorbol 12-myristate 13-acetate (PMA; Sigma -Aldrich Corporation, St Louis, MO, USA) 25 ng/ml and ionomycin (Calbiochem, Darmstadt, Germany) 1 µg/ml in 37°C, 5% CO_2_ for 4 hours. The cells were centrifuged for 300×g, 5 min and the supernatant was collected for enzyme-linked immunosorbent assay (ELISA). The cells were then further washed 3 times in PBS by centrifugation 300×g, 5 min, RT and collected for real-time polymerase chain reaction (PCR) analyses. Cells and supernatants were stored in −70°C until analysis.

The neutrophils were collected, washed and resuspended in PBS and NaCl and concentrated by a short centrifugation. The erythrocytes were lysed by a hypotonic solution and the neutrophils were washed with Krebs-Ringers glucose (KRG) without Ca^2+^ (0.1 mol/l NaCl, 5 mmol/l KCl, 1 mmol/l MgSO_4_, 2 mmol/l KH_2_PO_4_, 8 mmol/l Na_2_HPO_4_ and 10 mmol/l glucose). The neutrophils were then resuspended in RPMI-1640 with L-glutamine, 10% FBS and 100 U/ml penicillin 100 µg/ml streptomycin to a concentration of 5×10^6^ cells/ml and kept on ice. They were stimulated with PMA 25 ng/ml or interleukin (IL)-8 (Sigma -Aldrich Corporation, St Louis, MO, USA 10 ng/ml for 10 min in a 37°C water bath. The cells were then centrifuged for 300×g, 5 min, RT and the supernatant was collected for ELISA analyses. The neutrophils were further washed 3 times in PBS by centrifugation for 300×g, 5 min, RT and collected for real-time PCR analyses. Cells and supernatants were stored in −70°C until analysis.

### Determination of MMP-8, MMP-9, TIMP-1, TIMP-2 and myeloperoxidase (MPO) levels by ELISA

MMP-8, MMP-9, TIMP-1, TIMP-2 and myeloperoxidase (MPO) were measured in heparinized plasma, serum with activation clot and cell supernatants by ELISA (Quantikine® immunoassay, R&D systems, Minnneapolis, MN, USA). The MMP-8 ELISA measures both latent and active forms of the enzyme while the MMP-9 ELISA measures pro-MMP-9, active MMP-9 and MMP-9/TIMP-1 complexes. The lower limits of detection for MMP-8, MMP-9, TIMP-1, TIMP-2 and MPO were 0.02, 0.156, 0.08, 0.011 and 0.1 ng/ml, respectively. The interassay coefficients of variation (CV) were always <7%.

### Real-time PCR

Total RNA was isolated from separate fractions of PBMCs and neutrophils with RNeasy mini kit (Qiagen, Hilden, Germany) according to manufacturer's instructions. 0.73 µg RNA was reversed transcribed with high capacity cDNA reverse transcription kit with an RNAse inhibitor (Applied Biosystems, Foster City, CA, USA) according to manufacturer's instructions. cDNA (1 µL) was amplified by RT-PCR reactions with 1× TaqMan Fast Universal PCR Mastermix (Applied Biosystems, Foster City, CA, USA) in 96-well plates on an ABI 7500 Sequence Detector with SDS 1.3.1 software. The following TaqMan® Gene Expression Assay kits (Applied Biosystems) were used: MMP-8, Hs01029057_m1; MMP-9, Hs00957562_m1; TIMP-1, Hs00171558_m1; TIMP-2, Hs00234278_m1. Eukaryotic 18S rRNA (Part number: 4352930E) with an amplicon length of 187 bp served as endogenous control. The amount of expressed gene was calculated relative to the amount of rRNA in each sample. Standards were used to create a standard curve in each run according to the standard curve method in user bulletin no 2 (Applied Biosystem, Foster City, CA, USA). Each sample was run in duplicates and a maximum deviation of 15% was allowed.

### Statistics

PASW Statistics 18 was used for statistical analyses. For clinical and laboratory characteristics, data are presented as median (inter-quartile range). The statistical significance of any difference between patients and controls was tested by using Mann-Whitney U-test. Chi-square test was used for nominal data. Differences within the groups were analyzed using Wilcoxon signed-rank tests. Correlation analysis was performed using Spearman's rank correlation method. A p-value<0.05 was considered statistically significant while a p-value<0.1 was considered a trend.

## Results

### Clinical and laboratory characteristics of subjects

Clinical characteristics and laboratory variables of all patients and controls are outlined in Table 1. The waist circumference was significantly higher, and there were more current smokers among patients. The total number of white blood cells was also significantly increased in the patient group depending on a significant increase in neutrophils, but not PBMCs. All patients were using low-dose aspirin and various combinations of nitrates, beta-blockers and/or calcium antagonists. Seventy-one % of them had hypertension and 16% had diabetes. The majority of the patients (82%) had a normal left ventricular systolic function, while 14% had a mild and 4% had a severe ventricular dysfunction. Twenty-four % of the patients had a history of prior myocardial infarction and/or coronary revascularization. Eighty-six % of the patients were on long-term therapy with statin. The characteristics of the 10 patients and 10 controls in the subpopulations selected for ex vivo studies are shown in Table 2.

**Table 1:** Characteristics of patients with stable angina pectoris (SA) and controls in the whole cohort.

	Patients	Controls	p
Age, years	63 (57–71)	63 (58–71)	NS
Male/female	34/10	34/13	NS
Current smokers, n (%)	8 (18)	1 (2)	<0.05
Waist circumference, cm	101 (94–107)	95 (88–98)	<0.001
Total cholesterol, mmol/l	4.6 (4.0–5.4)	5.7 (4.9–6.6)	<0.001
LDL cholesterol, mmol/l	2.4 (2.1–3.4)	3.6 (2.8–4.2)	<0.001
HDL cholesterol, mmol/l	1.2 (1.1–1.4)	1.5 (1.2–1.7)	<0.001
Triglycerides, mmol/l	1.4 (1.1–1.7)	1.1 (0.93–1.6)	<0.05
Creatinine, µmol/l	92 (83–104)	88 (78–99)	NS
White blood cells, cell/µl	6600 (5100–8400)	5500 (4600–6400)	0.001
Diabetes, n (%)	7(16)	0 (0)	0,005
Hypertension, n (%)	32 (73)	0 (0)	<0,001
Statin, long-term treatment, n (%)	36 (86)	0 (0)	<0,001

Data are given as median (inter-quartile range).

**Table 2:** Characteristics of the subpopulations of patients with stable angina pectoris (SA) and controls selected for the ex vivo assays.

	Patients	Controls	p
Age, years	60 (56–68)	63 (57–69)	NS
Male/female	6/4	6/4	NS
Current smokers, n (%)	3 (30)	0 (0)	NS
Waist circumference, cm	101 (95–103)	95 (88–98)	0,062
Total cholesterol, mmol/l	4,2 (3,8–4,3)	6,3 (5,5–6,8)	0,001
LDL cholesterol, mmol/l	2,3 (1,8–2,5)	3,9 (3,4–4,7)	0,002
HDL cholesterol, mmol/l	1,1 (1,1–1,4)	1,7 (1,5–1,8)	0,01
Triglycerides, mmol/l	1,4 (1–1,5)	1,2 (0,97–1,9)	NS
Creatinine, µmol/l	83 (67–89)	73 (57–82)	NS
White blood cells, cell/µl	7100 (5400–8600)	5700 (4700–6400)	NS
Diabetes, n (%)	3 (30)	0 (0)	NS
Hypertension, n (%)	8 (80)	0 (0)	0,001
Statin, long-term treatment n (%)	10 (100)	0 (0)	<0,001

Data are given as median (inter-quartile range).

### MMP-8 and MMP-9

The levels of MMP-8 and MMP-9 in plasma, serum and cell supernatants from neutrophils are presented in Table 3. The levels in plasma did not differ between patients and controls, neither did the serum levels. The spontaneous release of MMP-9 into the neutrophil supernatant tended to be higher in patients compared with controls and became statistically significant after IL-8 stimulation. However, after PMA stimulation the neutrophil release of MMP-9 was significantly lower in the patient group. The release of MMP-9 from both untreated and stimulated PBMC was negligible (data not shown). The release of MMP-8 was similar in non-treated and IL-8-treated neutrophils, but increased markedly upon PMA-stimulation in both patients and controls. MMP-8 in PBMC or platelet supernatants was not detectable (data not shown).

**Table 3:** MMP-8 and MMP-9 levels (ng/ml) in plasma, serum and neutrophil supernatants.

		n (pat/co)	Patients	Controls	p
**MMP-8**	Plasma	44/47	3.1 (1.5–3.5)	3.1 (2.2–5.7)	NS
	Serum	10/10	12.4 (5.2–39)	8.1 (5.7–19)	NS
	Spontaneous release	10/10	8.5 (4.6–10.4)	7.3 (5.9–9)	NS
	IL-8-induced release	10/10	10.0 (5.7–15.4)	8.9 (7–10.3)	NS
	PMA-induced release	10/10	142 (96–192)	174 (133–190)	NS
**MMP-9**	Plasma	44/47	67 (38–107)	53 (43–85)	NS
	Serum	10/10	438 (275–1049)	388 (215–608)	NS
	Spontaneous release	10/10	126 (100–184)	93 (90–142)	0.07
	IL-8-induced release	10/10	306 (214–333)	208 (175–289)	<0.05
	PMA-induced release	10/10	1261 (1104–1441)	1557 (1382–1708)	<0.05

Data are given as median (inter-quartile range).

### TIMP-1 and TIMP-2

The levels of TIMP-1 and TIMP-2 in plasma, serum and cell supernatants are presented in Table 4. The levels of TIMP-1 in plasma or serum did not differ between patients and controls. TIMP-1 was not detectable in neutrophil supernatants. In PBMC supernatants, it was detected after 19 h in culture without any significant differences between patients and controls (data not shown). The plasma levels of TIMP-2 were significantly lower in patients compared with controls. The serum levels of TIMP-2 also tended to be lower in the patient group. Upon IL-8 or PMA stimulation, the release of TIMP-2 from neutrophils increased by a similar extent in patients and controls. The release of TIMP-2 from PBMC was similar in patients and controls regardless of cell stimulation.

**Table 4:** TIMP-1 and TIMP-2 levels (ng/ml) in plasma, serum, neutrophil and PBMC supernatants.

		n (pat/co)	Patients	Controls	p
**TIMP-1**	Plasma	44/47	87 (78–95)	92 (80–103)	NS
	Serum	10/10	193 (168–213)	180 (151–191)	NS
	Neutrophil release, untreated	10/10	ND	ND	
	Neutrophil release, IL-8	10/10	ND	ND	
	Neutrophil release, PMA	10/10	ND	ND	
	PBMC release, untreated 4 h	10/10	ND	ND	
	PBMC release, PMA 4 h	10/10	ND	ND	
**TIMP-2**	Plasma	44/47	70 (65–77)	79 (72–83)	<0.01
	Serum	10/10	77 (72–86)	85 (80–87)	0.08
	Neutrophil release, untreated	10/10	1.3 (1.2–1.5)	1.3 (1–1.4)	NS
	Neutrophil release, IL-8	10/10	2.4 (2–2.9)	2.2 (2–2.4)	NS
	Neutrophil release, PMA	10/10	10.2 (8–11.6)	11.1 (10–11.7)	NS
	PBMC release, untreated 4 h	10/10	1.0 (0.8–1.2)	1.0 (0.9–1.2)	NS
	PBMC release, PMA 4 h	10/10	1.5 (1.1–1.8)	1.5 (1.3–1.7)	NS

ND = Non Detectable.

Data are given as median (i-q range).

### MPO

The levels of MPO in plasma, serum and cell supernatants are presented in Table 5. The levels of MPO in plasma and serum did not differ between patients and controls. In SA patients, MPO was significantly higher in serum than in plasma, whereas in controls, the serum and plasma levels were similar. The spontaneous release of MPO from neutrophils tended to be higher in patients than in controls. Upon PMA stimulation, the release of MPO increased significantly in the control group, whereas there was no statistically significant increase in the patient group.

**Table 5:** MPO levels (ng/ml) in plasma, serum and neutrophil supernatants.

		n (pat/co)	Patients	Controls	p
MPO	Plasma	44/47	325 (196–494)	351 (265–486)	NS
	Serum	10/10	580 (316–909)^a^	338 (193–468)	NS
	Spontaneous release	10/10	1237 (1140–1850)	1046 (858)	0.09
	PMA-induced release	10/10	1452 (1270–1950)	1526 (891–1740)^b^	NS

a p<0.01 compared with plasma in patients.

b p<0.001 compared with spontaneous release in neutrophils from controls.

Data are given as median (i-q range).

### Real-time PCR

**Figure 1:**
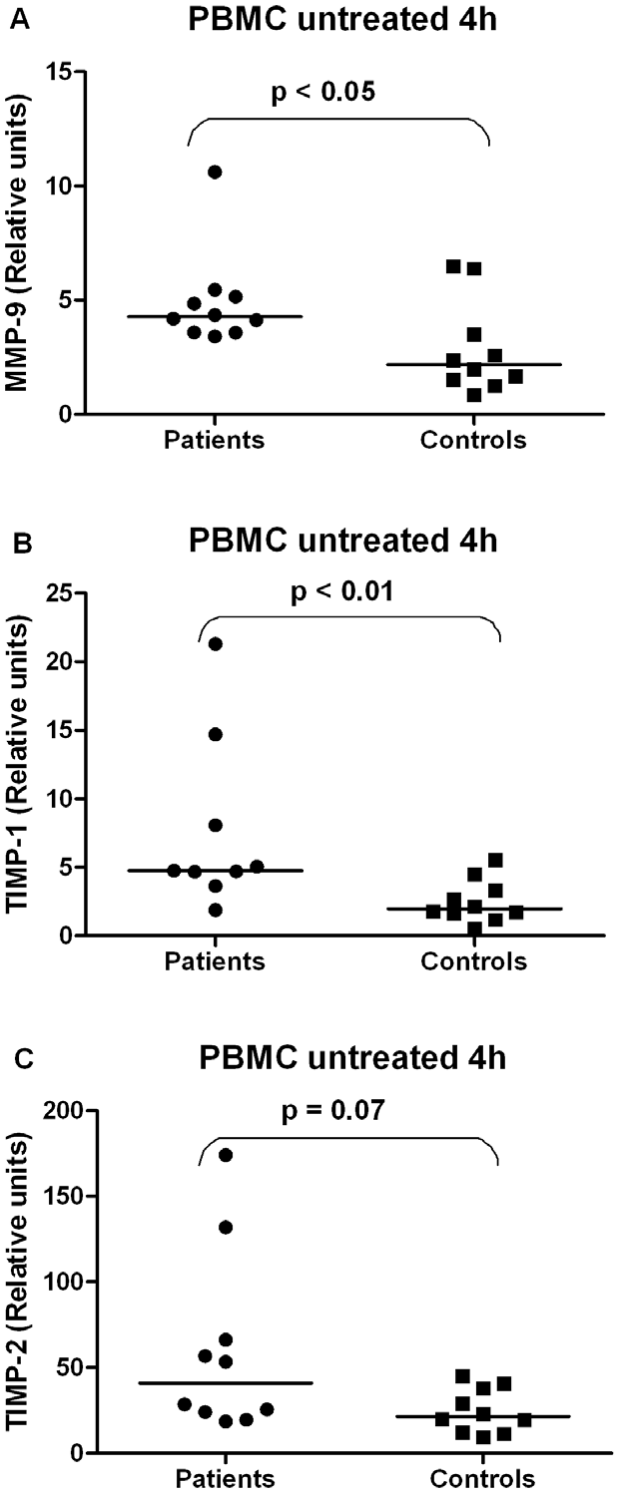
Gene expression of MMP-9 and TIMP-1 in PBMC. MMP-9 (A) and TIMP-1 (B) mRNA expression was higher in PBMC of CAD patients compared with controls (p<0.05 and 0.01, respectively). The expression of TIMP-2 (C) tended to be increased in PBMC of patients (p = 0.07).

The mRNA expression of MMP-9 and TIMP-1 in PBMC (left untreated for 4 h) was significantly higher in patients compared with controls (Figure 1a and b) while the TIMP-2 mRNA levels in PBMC tended to be increased in the patient group (Figure 1c). After 4 h stimulation, the mRNA expression of MMP-9 and TIMP-1 in PBMC increased but without any significant differences between patients and controls (data not shown). On the other hand, PBMC from controls showed an increase in TIMP-2 mRNA after 4 h stimulation reaching similar levels as PBMC from patients. In neutrophils, the mRNA expression of MMP-9, TIMP-1 and TIMP-2 did not differ between patients and controls neither before nor after stimulation with IL-8 or PMA (data not shown). MMP-8 mRNA was not detectable in either neutrophils or PBMC.

### Correlations

The levels of MMP-8 and MMP-9 in plasma did not correlate with any clinical characteristics, such as waist circumference or smoking; neither did the plasma levels correlate with lipid or creatinine levels. MMP-8 and MMP-9 in plasma were correlated to the white blood cell count, *r* = 0.24 and 0.30 respectively, p<0.05. In addition, MMP-9 was correlated with the neutrophil count (*r* = 0.28, p<0.05), but not with the PBMC count. In plasma, MMP-8 and MMP-9 were intercorrelated (r = 0.87, p<0.001) and they were also highly correlated to MPO (both r = 0.80, p<0.001). TIMP-1 in plasma did not show any significant correlations with clinical or laboratory parameters whereas TIMP-2 in plasma was inversely correlated to waist circumference (*r* = −0.21, p<0.05) and positively related to HDL cholesterol levels (*r* = 0.22, p<0.05). HDL levels were inversely correlated to waist circumference (r = −0.37, p<0.001) and when both variables were entered into a multiple linear regression model, their significant correlation to TIMP-2 was lost. Patients and controls were grouped together for all correlation analyses.

## Discussion

Among peripheral blood cells, the neutrophils were found to be a dominant source of MMP-8 and MMP-9, as assessed by short-term release assays. This was not an unexpected finding since previous work has shown that neutrophils are responsible for the rapid release of MMPs that occurs in blood upon stimulation with endotoxin or proinflammatory cytokines [31]–[33]. However, in SA patients, the neutrophils released more MMP-9 in response to IL-8 than neutrophils from controls. These results are in line with a study by Tayebjee et al [20] who showed increased amounts of intracellular neutrophil MMP-9, as assessed by flow cytometry, in patients with SA compared with healthy controls. Since the neutrophil release of MMP-9 is assumed to serve as a sensitive and early marker of neutrophil activation [32], the findings may indicate a persistent neutrophil activation in the SA patients. The appropriate use of MMP-9 as a neutrophil activation marker was strengthened by its high correlation to MPO, another principal enzyme released upon neutrophil activation [31]. In a previous study, we also demonstrated an increased number of circulating neutrophil-platelet aggregates in patients with SA which further supports the presence of activated neutrophils in CAD [34]. On the other hand, we and others have demonstrated an impaired neutrophil response to intense stimulation in SA patients including a reduced capacity to generate reactive oxygen species in response to strong stimuli [35], [36]. IL-8 may be regarded as a physiological stimulator of neutrophils while compounds like PMA are more likely to trigger maximal degranulation. When the neutrophils from patients were stimulated with PMA in the present study, they released significantly lower amounts of MMP-9 compared with controls. The extensive statin treatment in stable CAD patients may at least partly explain the suboptimal response of neutrophils to a strong stimulus. In a randomized clinical study, the endotoxin-induced increase in neutrophil oxidative burst was markedly attenuated by simvastatin [37]. The treatment with low-dose aspirin may also exert suppressive effects on neutrophils. Aspirin has been shown to attenuate the inflammatory response in neutrophils, e.g. by decreasing the transmigration capacity and inhibiting the delayed apoptosis of cells [38], [39].

In unstimulated PBMC, the mRNA expression of MMP-9 was found to be significantly increased in SA patients compared with controls. The expression of MMPs in human mononuclear cell subsets has been systematically analyzed in a previous study by Bar-Or et al [24] showing that MMP-9 mRNA was particularly abundant in monocytes compared with B and T cells. However, our findings of upregulated MMP-9 mRNA in SA patients is not in agreement with two recent studies of CAD patients. When Brunner et al [14] compared MMP-9 mRNA in purified unstimulated monocytes, they found no differences between SA patients (n = 18) and controls (n = 16) but a significant increase in patients with acute coronary syndrome. Likewise, Fang et al [25]cultured PBMC for 24 h and reported no difference in MMP-9 mRNA between SA patients (n = 8) and controls (n = 12) but a 2-fold increase in patients with acute coronary syndrome. One possible reason for our discrepant results regarding SA patients may be an increased ischemic burden in our patient group, i.e. they were all referred for coronary angiography due to disabling angina pectoris. In vitro, a variety of proinflammatory and atherogenic factors including cytokines and oxidized low-density lipoproteins has been shown to upregulate MMP-9 expression in monocytes [24], [40]. Our findings suggest that this may be relevant also in vivo under clinically stable conditions.

Not only MMP-9 mRNA, but also TIMP-1 mRNA, was increased in untreated PBMC from patients. Likewise, the transcript levels of TIMP-2 tended to be increased in the PBMC of patients. As previously shown, TIMP-1 and, to a lesser extent TIMP-2, is constitutively expressed and secreted by monocytes [14], [24], [28], [41]. The concomitant increase of MMP-9 and TIMP mRNA in PBMC from patients was not a surprising finding since the expression of proteases in many physiological settings goes along with the simultaneous expression of their inhibitors. Upon stimulation with PMA, the induction of MMP-9 synthesis by mononuclear cells has been shown to be accompanied by production of TIMP-1 [28]. Despite increased mRNA expression, only negligible amounts of MMPs and TIMPs were released by PBMC upon 4 h treatment with PMA. PMA has been shown to be a potent inducer of MMP and TIMP in experimental studies of blood-derived mononuclear cells [28], [41]. Still, it is possible that we did not use the appropriate stimulation conditions to allow protein secretion.

In agreement with a number of previous studies [12]–[14], [20], there were no significant differences in circulating levels of MMP-9 between SA patients and controls. Neither did the plasma or serum levels of MMP-8 differ between patients and controls which is in contrast to previous studies [11], [18] that have shown raised plasma MMP-8 in SA patients. As expected, serum levels were markedly increased compared with plasma levels probably due to an extensive degranulation of neutrophils in the serum sample. In accordance with this, the time-dependent release of other neutrophil mediators, such as leukotriene B_4_ has been seen to occur during serum preparation [42].

The serum and plasma concentrations of TIMP-1 were similar in patients and controls while TIMP-2 in plasma was significantly lower in the patient group. In line with our results, two earlier reports on TIMP-2 have shown lower levels in SA patients compared with controls [13], [15] while Tayebjee et al [16] demonstrated increased levels of TIMP-2 in patients. Compared with TIMP-1, TIMP-2 showed a significant relationship to metabolic factors like waist circumference and HDL concentrations. A differential relationship of TIMP-1 and TIMP-2 to circulating markers of inflammation and hemostasis has been reported previously [43]. The imbalance between MMP-9, TIMP-1 and TIMP-2 expression in the atherosclerotic plaque may be crucial for its transformation into an unstable, rupture-prone state. Interestingly, TIMP-2 has been shown to be more effective than TIMP-1 in inhibiting MMP-9 [44]. We can only speculate that the plasma levels reflect, at least in part, the levels of TIMP-2 in atherosclerotic tissue. In a previous study on patients who had undergone successful carotid artery endarterectomy, a decreased production of TIMP-2 in unstable carotid lesions was associated with decreased levels in plasma [26].

There are certain limitations of our study. The study population was small and results should therefore be interpreted with caution, neither is it possible to evaluate the prognostic significance of the results. The control group was a clinically healthy population without any chronic medication. However, they were not angiographically examined and the possibility of subclincal atherosclerosis can not be ruled out.

To conclude, our findings indicate that measurements of MMPs and TIMPs in plasma may be of limited use in the evaluation of inflammatory status in CAD patients. Despite “normal” plasma levels, the leukocyte-derived MMP-9 and TIMP-1 were significantly altered in patients with symptomatic and angiographically verified CAD. First, the neutrophils released significantly more MMP-9 upon moderate stimulation which indicates the presence of neutrophils that are more prone to stimulation. Second, the increased mRNA levels of MMP-9 and TIMP-1 in PBMC suggest that these cells might also be preactivated with an increased transmigratory capacity. However, so far we can only theorize about the effects of increased leukocyte-derived MMP-9. Its potential clinical implications remain to be clarified in future studies.
